# Wild-type FUS corrects ALS-like disease induced by cytoplasmic mutant FUS through autoregulation

**DOI:** 10.1186/s13024-021-00477-w

**Published:** 2021-09-06

**Authors:** Inmaculada Sanjuan-Ruiz, Noé Govea-Perez, Melissa McAlonis-Downes, Stéphane Dieterle, Salim Megat, Sylvie Dirrig-Grosch, Gina Picchiarelli, Diana Piol, Qiang Zhu, Brian Myers, Chao-Zong Lee, Don W Cleveland, Clotilde Lagier-Tourenne, Sandrine Da Cruz, Luc Dupuis

**Affiliations:** 1 Mécanismes centraux et périphériques de la neurodégénérescence, Centre de Recherches en Biomédecine, Université de Strasbourg, Inserm, UMR-S1118, Strasbourg, France; 2 Ludwig Institute for Cancer Research, University of California at San Diego, La Jolla, USA; 3 VIB-KU Leuven Center for Brain and Disease Research, Department of Neurosciences, KU Leuven, Leuven, Belgium; 4 Department of Neurology, Massachusetts General Hospital, The Sean M. Healey and AMG Center for ALS at Mass General, Harvard Medical School, Boston, MA USA; 5 Broad Institute of Harvard University and MIT, Cambridge, MA USA

**Keywords:** Amyotrophic lateral sclerosis, Fronto-temporal dementia, Mouse models, RNA-binding proteins, FUS, Autoregulation, Therapy

## Abstract

Mutations in FUS, an RNA-binding protein involved in multiple steps of RNA metabolism, are associated with the most severe forms of amyotrophic lateral sclerosis (ALS). Accumulation of cytoplasmic FUS is likely to be a major culprit in the toxicity of *FUS* mutations. Thus, preventing cytoplasmic mislocalization of the FUS protein may represent a valuable therapeutic strategy. FUS binds to its own pre-mRNA creating an autoregulatory loop efficiently buffering FUS excess through multiple proposed mechanisms including retention of introns 6 and/or 7. Here, we introduced a wild-type *FUS* gene allele, retaining all intronic sequences, in mice whose heterozygous or homozygous expression of a cytoplasmically retained FUS protein (*Fus*^∆NLS^) was previously shown to provoke ALS-like disease or postnatal lethality, respectively. Wild-type FUS completely rescued the early lethality caused by the two *Fus*^∆NLS^ alleles, and improved the age-dependent motor deficits and reduced lifespan caused by heterozygous expression of mutant FUS^∆NLS^. Mechanistically, wild-type FUS decreased the load of cytoplasmic FUS, increased retention of introns 6 and 7 in the endogenous mouse *Fus* mRNA, and decreased expression of the mutant mRNA. Thus, the wild-type *FUS* allele activates the homeostatic autoregulatory loop, maintaining constant FUS levels and decreasing the mutant protein in the cytoplasm. These results provide proof of concept that an autoregulatory competent wild-type FUS expression could protect against this devastating, currently intractable, neurodegenerative disease.

**Supplementary Information:**

The online version contains supplementary material available at 10.1186/s13024-021-00477-w.

## Background

Amyotrophic lateral sclerosis (ALS), the major adult onset motor neuron disease [1, 2], is characterized by a progressive paralysis leading to death within a few years after onset. Mutations in *FUS* cause the most severe cases of ALS, with young onset and rapid disease progression [3, 4]. *FUS* mutations are clustered in the C-terminal region of the protein, carrying a PY-nuclear localization sequence (NLS), responsible for its nuclear import. Truncating mutations have been described in ALS families, leading to complete loss of the PY-NLS, and cytoplasmic aggregation of FUS [5, 6]. Studies in mouse models have demonstrated that cytoplasmic accumulation of FUS provokes motor neuron degeneration [7–12]. Indeed, heterozygous *Fus* knock-in mice with ALS-like truncating mutations develop mild, late onset muscle weakness and motor neuron degeneration, while haploinsufficient *Fus* knock-out mice do not show ALS related symptoms [10–12]. A successful therapeutic strategy for *FUS*-ALS may lie in reduction of the cytoplasmic FUS content, to avoid its toxic effects.

FUS levels are regulated by other RNA-binding proteins [13, 14] and are tightly controlled by autoregulatory mechanisms [14–16]. Indeed, the addition of more than 20 copies of the complete human *FUS* gene to the mouse genome only slightly increases FUS protein levels, and does not lead to phenotypic consequences [8], showing the efficacy of this buffering system of FUS levels. Contrastingly, the saturation of FUS autoregulation, through overexpression of cDNA driven, autoregulatory incompetent, FUS expression, is highly toxic to neurons [9, 17]. FUS autoregulation appears to involve at least three possible mechanisms, including exon skipping [15], intron retention [14] and microRNA [16], and recent evidence suggested that the major autoregulatory mechanism was retention of introns 6 and 7 [14]. Here, we tested the hypothesis that the expression of a wild-type *FUS* gene, carrying all regulatory elements necessary for autoregulation would engage autoregulation of the mutation carrying RNA, and subsequently decrease accumulation of FUS in the cytoplasm.

## Results

### Wild-type ***FUS*** transgene rescues lethality and motor defects in ***Fus***^∆NLS^ mice

**Fig. 1:**
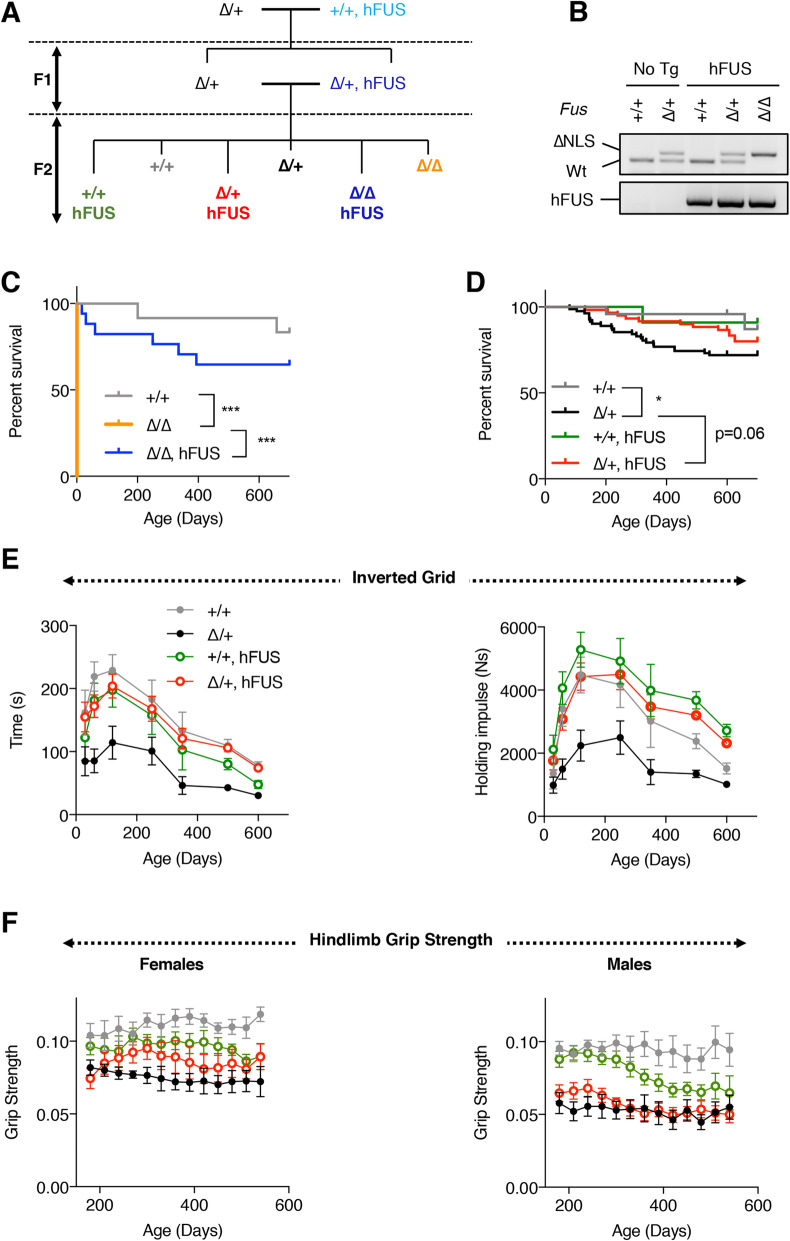
hFUS transgene rescues lethality and motor deficits in *Fus*^∆NLS^ mice. **A**: Scheme of the breeding strategy. **B**: Representative genotyping results of 5 mice at 1 month of age. **C**-**D**: Kaplan Meier survival curve of the different genotypes either homozygous (**C**) or heterozygous for the ∆NLS mutation (**D**). Note that all *Fus*^∆NLS/∆NLS^ mice die at birth, unless carrying a hFUS transgene. *, *p* < 0.05 Log Rank test; ***, *p* < 0.001 Log rank test. **E**: Age-dependent changes in the mean hanging time (s) and holding impulse (Ns) in the four-limb wire inverted grid test in *Fus*^+/+^ (+/+), and *Fus*^*ΔNLS*/+^ (∆/+) mice with or without hFUS transgene. *N* = 10–28 per group. Mixed effect analysis, with 3 factors (Age, ∆NLS genotype and hFUS genotype). *P < 0.001* for ∆NLS genotype, *P < 0.001* for age, *P < 0.001* for hFUS genotype. A significant protective interaction is observed between ∆NLS and hFUS genotypes (*p* = 0.0216, and *p = 0.0366).* Only 4 groups out of 5 are shown here for clarity. The whole dataset is shown in Fig S1. **F**: Hindlimb grip strength in female and male mice. Mixed effect analysis, with 3 factors (Age, ∆NLS genotype and hFUS genotype). For female mice, *P* < 0.001 for ∆NLS genotype, p = ns for age, *p* = ns for hFUS genotype. A significant protective interaction is observed between ∆NLS and hFUS genotypes (*p* = 0.0131). For male mice, *P* < 0.001 for ∆NLS genotype, *p* = ns for age, *p* = ns for hFUS genotype. No significant protective interaction is observed between ∆NLS and hFUS genotypes (*p* = 0.0512)

Human wild-type FUS transgenic mice (hFUS mice) expressing human *FUS* gene including its own human *FUS* promoter obtained from a BAC [8] were crossed with *Fus*^∆NLS^ mice [11] in a two-round mating (Fig. 1 A). As previously described, *Fus*^∆NLS/∆NLS^ mice (in absence of hFUS) die within the first hours after birth [11] and no homozygous mutant *Fus*^∆NLS^ mice were obtained at 1 month of age in the absence of hFUS (Fig. 1B). Contrastingly, expression of hFUS transgene completely rescued lethality of homozygous *Fus*^∆NLS/∆NLS^ mice until adulthood (Fig. 1 C). However, rescued homozygous *Fus*^∆NLS/∆NLS^ mice displayed higher lethality throughout adulthood than wild-type littermate animals (Fig. 1 C). Increased adult lethality was also observed in *Fus*^∆NLS/+^ mice, with about a 30 % of death rate before 600 days of age (p = 0.0398, log rank, *Fus*^+/+^ vs. *Fus*^∆NLS/+^), consistent with findings reported in another heterozygous knock-in model [10]. Nonetheless, most *Fus*^∆NLS/+^/hFUS mice survived until this age, and their survival rate was indistinguishable from non-transgenic normal mice (*p* = 0.33 *Fus*^+/+^ vs. *Fus*^∆NLS/+^/hFUS) or from single hFUS transgenic mice (Fig. 1D). The mild, late onset, muscle weakness observed in *Fus*^∆NLS/+^ mice using inverted grid test [12], was rescued in *Fus*^∆NLS/+^/hFUS and in *Fus*^∆NLS/∆NLS^/hFUS mice (Fig. 1E and Fig. S1A). Furthermore, hindlimb grip strength deficits associated with expression of *Fus*^∆NLS/+^ were mildly and transiently improved in *Fus*^∆NLS/+^/hFUS females (Fig. 1 F) but not in males (Fig. 1 F). Indeed, in this test, the performance of hFUS transgenic mice decreased significantly in males after 10 months of age, thus confounding a potential protection (Fig. 1 F and Fig. S1A). These protective effects were not caused or modified by changes in body weight as there were no significant changes in body weight across genotypes before 200 days of age. After this age, only *Fus*^∆NLS/∆NLS^/hFUS mice showed a mildly decreased body weight as compared to the wild-type and *Fus*^∆NLS/+^ mice (Fig. S1B). Thus, wild-type human FUS significantly rescued lethality and, at least partially, motor deficits associated with cytoplasmically retained mutant FUS^∆NLS^ protein.

### Wild-type ***FUS*** transgene decreases cytoplasmic accumulation of FUS in ***Fus***^∆NLS^ mice

**Fig. 2:**
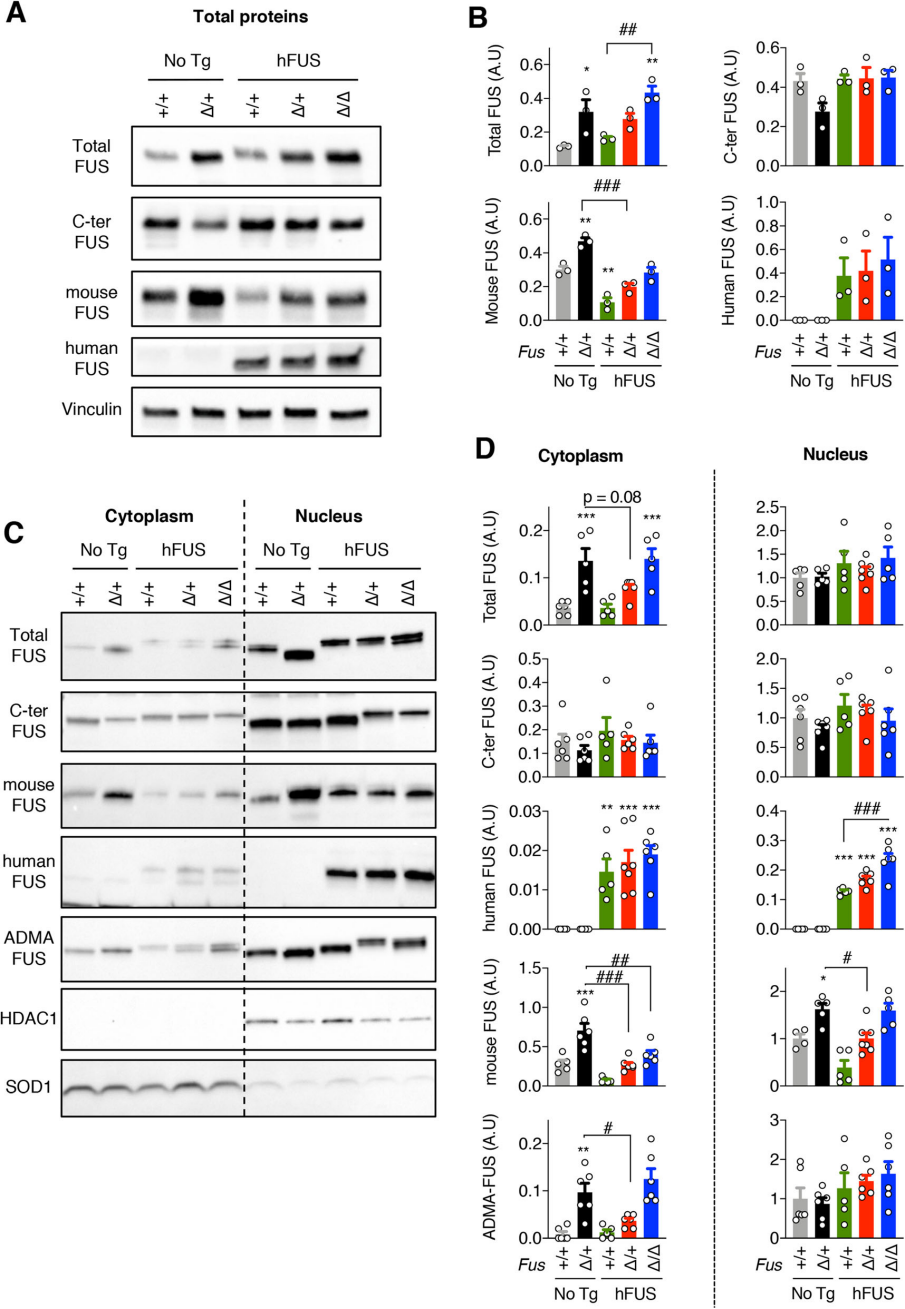
hFUS transgene corrects abnormal levels of FUS protein in *Fus*^∆NLS^ mice. **A**: Immunoblot analysis of FUS protein in total extracts from *Fus*^+/+^ (+/+) and *Fus*^*ΔNLS*/+^ (∆/+) brains with or without hFUS transgene and of *Fus*^*ΔNLS*/∆NLS^ mice (∆/∆) with hFUS transgene at 22 months of age. Representative results using different antibodies revealing total FUS, the C-terminal (C-ter) NLS, mouse FUS, and human FUS. Vinculin was used as loading controls. Note that these immunoblots were performed on different membranes to avoid cross reaction between different antibodies and one representative Vinculin blot is shown. Uncropped western blots and their corresponding Vinculin western blots are provided in Source data. **B**: Quantification of immunoblotting experiments of panel **A**. Quantification of total, C-ter, mouse, human and ADMA-FUS protein levels in cytoplasmic and nuclear fractions of the indicated genotypes. *N* = 4–8. *p < 0.05, ***p < 0.001vs *Fus*^*+/+*^, #, *p* < 0.05 and ###, *p* < 0.001 vs. indicated genotype by ANOVA followed by Tukey. **C**: Immunoblot analysis of FUS protein subcellular localization in cortex of *Fus*^+/+^ (+/+) and *Fus*^*ΔNLS*/+^ (∆/+) mice with or without hFUS transgene and of *Fus*^*ΔNLS*/∆NLS^ mice (∆/∆) with hFUS transgene at 1 month of age. Representative results using different antibodies revealing total FUS, the C-terminal (C-ter) NLS, mouse FUS, human FUS and asymmetrically dimethylated arginine FUS (ADMA-FUS). SOD1 and HDAC1 are used as loading controls for cytoplasmic and nuclear protein extracts fractions, respectively. Note that these immunoblots were performed on different membranes to avoid cross reaction between different antibodies. Uncropped western blots and corresponding stain free gels are provided in Source data. **D**: Quantification of western blotting experiments of panel **C**. Quantification of total, C-ter, mouse, human and ADMA-FUS protein levels in cytoplasmic and nuclear fractions of the indicated genotypes. *N* = 4–8. *p < 0.05, ***p < 0.001vs *Fus*^*+/+*^, #, *p* < 0.05 and ###, *p* < 0.001 vs. indicated genotype by ANOVA followed by Tukey

We then asked whether hFUS transgene altered levels of FUS in *Fus*^∆NLS^ mice. Consistent with previous results [18], total FUS levels increased in *Fus*^∆NLS/+^ mouse brains as compared to *Fus*^+/+^ mice, and in *Fus*^∆NLS/+^/hFUS as compared to single hFUS transgenic mice (Fig. 2 A-B). Consistent with previous results, the hFUS transgene on its own did not further increase total FUS proteins in wild type or *Fus*^∆NLS/+^ mice. The increase observed in *Fus*^∆NLS/+^ mice was not detected when an antibody targeting the NLS sequence (C-term FUS), absent from the FUS^∆NLS^ protein, was used, but was even more evident using an antibody targeting selectively mouse FUS (Fig. 2 A-B and Source data for uncropped western blots). This increase in mouse FUS was normalized by the hFUS transgene. Human FUS levels remained unchanged across the three genotypes carrying hFUS.

**Fig. 3:**
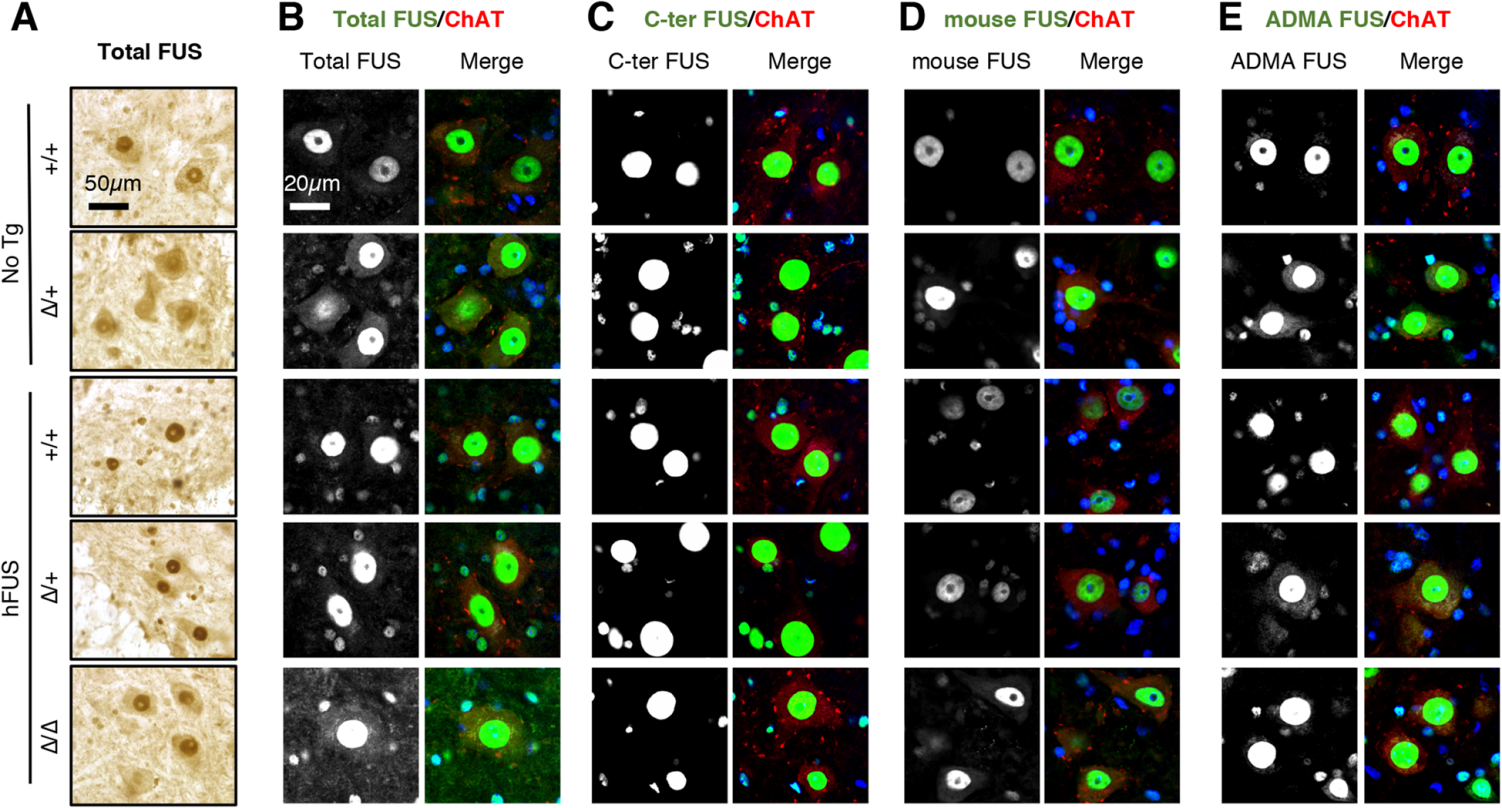
hFUS transgene corrects abnormal cytoplasmic accumulation of FUS in *Fus*^∆NLS^ motor neurons. **A**: FUS immunohistochemistry in the spinal cord ventral horn at 22 months of age of mice with the indicated genotypes labelled as in previous figures. Note that diffuse FUS cytoplasmic staining, obvious in *Fus*^*ΔNLS*/+^ mice, is rescued by the hFUS transgene. Scale bar: 50 μm. **B-E**: Double immunostaining for the motorneuronal marker ChAT (red), nuclei (blue) and either total FUS (**B**), C-terminal FUS (**C**), mouse FUS (**D**) or ADMA-FUS (**D**) in the spinal cord ventral horn at 22 months of age. Scale bar: 20 μm

We then asked whether increased FUS cytoplasmic levels were also rescued by the hFUS transgene and performed subcellular fractionation to obtain nuclear and cytoplasmic fractions. Indeed, and as expected [11, 12], cytoplasmic FUS levels were elevated by five-fold in cerebral cortex of *Fus*^∆NLS/+^ mice as compared to corresponding wild-type mice (Fig. 2 C-D and Source data for uncropped western blots) demonstrating that this increase is related to the mislocalization of the mutant protein. Importantly, the increase in mouse FUS in cytoplasmic fractions of *Fus*^∆NLS/+^ mice, was normalized by the hFUS transgene (Fig. 2 C). Contrastingly, nuclear FUS levels were similar in all genotypes, irrespective of the presence of the *Fus*^∆NLS^ mutation or that of the hFUS transgene. Human FUS levels were increased in *Fus*^∆NLS/∆NLS^ mice carrying a hFUS transgene, likely compensating for the loss of nuclear FUS of mouse origin. In spinal cord sections, *Fus*^∆NLS/+^ neurons displayed a mixed cytoplasmic and nuclear FUS staining, that was prevented by the hFUS transgene (Fig. 3 A), and this was also observed in motor neurons using an antibody detecting total FUS using double FUS/ChAT immunofluorescence (Fig. 3B). No cytoplasmic staining was observed when using a C-terminal antibody (Fig. 3 C), further confirming that the cytoplasmic staining is derived from mutant FUS protein. Indeed, specific immunolabelling of mouse FUS showed decreased overall signal in mice with hFUS transgene, and loss of cytoplasmic staining in *Fus*^∆NLS/+^ /hFUS motor neurons (Fig. 3D). Interestingly, we observed significant nuclear staining for mouse FUS in *Fus*^∆NLS/∆NLS^/hFUS motor neurons despite the lack of NLS in mouse FUS in this genotype (Fig. 3D). Accumulation of cytoplasmic asymmetrically dimethylated (ADMA) FUS is a feature of *FUS*-ALS [5, 6, 19] patients which was recapitulated in the *Fus*^∆NLS/+^ mice, as we previously reported [12]. Here, this significant increase in ADMA-FUS detected in *Fus*^∆NLS/+^ cytoplasmic fractions, was largely prevented by the hFUS transgene in *Fus*^∆NLS/+^/hFUS mice (Fig. 3 C-D), but not in *Fus*^∆NLS/∆NLS^/hFUS mice. While ADMA-FUS immunoreactivity was clearly detected in the cytoplasm of *Fus*^∆NLS/+^ motor neurons, expression of the hFUS transgene in *Fus*^∆NLS/+^/hFUS led to reduced ADMA-FUS immunoreactivity signal in *Fus*^∆NLS/+^ mice (Fig. 3E). It should be noted however, that motor neurons of *Fus*^∆NLS/∆NLS^/hFUS mice still displayed residual amounts of cytoplasmic FUS (Fig. 3B, D, E). These results thus suggest that wild-type hFUS restores aberrant FUS nearly to normal levels but does not completely abolish FUS mislocalization.

### Wild-type ***FUS*** transgene activates autoregulation of mutant ***Fus*** to decrease mutant FUS protein

**Fig. 4:**
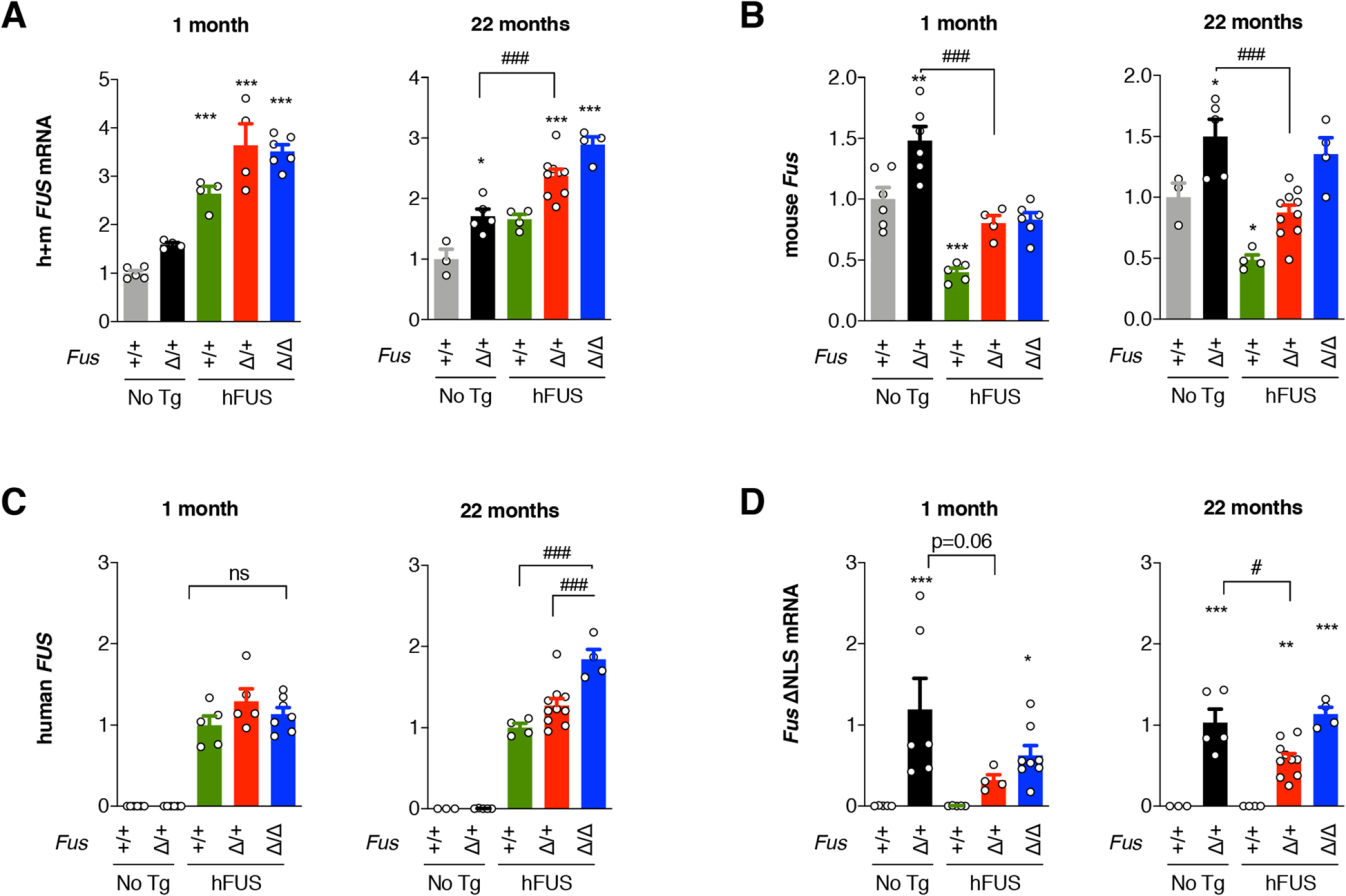
hFUS transgene downregulates endogenous *Fus* mRNA levels in the spinal cord. **A-D**: RT-qPCR results for total (human + mouse) mRNA encoding FUS (**A**), endogenous mouse *Fus* mRNA (**B**), human FUS transgene (**C**) and mutant *Fus* mRNA carrying the ∆NLS mutation (**D**) in spinal cord at 1 month (left) or 22 months (right) of age. Note that the hFUS transgene decreases expression of endogenous *Fus* gene and leads to decreased expression of mutant *Fus* mRNA at 1 and 22 months of age in the spinal cord. *N* = 4–8. *, *p* < 0.05, **p < 0.01, **p < 0.001 vs. *Fus*^*+/+*^, #, *p* < 0.05, ###, *p* < 0.001 vs. indicated genotype by ANOVA followed by Tukey

Consistent with the results of western blotting, total levels of mRNA encoding FUS (both endogenous mouse and human transgene derived) increased in *Fus*^∆NLS/+^ spinal cord, and were further elevated by the hFUS transgene in *Fus*^∆NLS/+^/hFUS and *Fus*^∆NLS/∆NLS^/hFUS spinal cord (Fig. 4 A) and frontal cortex (Fig S2). However, levels of endogenous *Fus* mRNA, that are increased in *Fus*^∆NLS/+^ mice, were corrected by hFUS transgene in 1-month old spinal cord (Fig. 4B) and frontal cortex (Fig. S2) of *Fus*^∆NLS/+^/hFUS and *Fus*^∆NLS/∆NLS^/hFUS animals, leading to accumulated mouse *Fus* mRNA levels close to those of endogenous *Fus* in normal non-transgenic mice. This restoration of mouse *Fus* mRNA levels by hFUS transgene was sustained through aging as observed in 22-month old *Fus*^∆NLS/+^/hFUS mice. Consistently, mutant *Fus*^*∆NLS*^ mRNA levels decreased in spinal cord and frontal cortex of *Fus*^∆NLS/+^/hFUS and *Fus*^∆NLS/∆NLS^/hFUS animals compared to the *Fus*^∆NLS/+^ mice (Fig. 4D and Fig. S2), while human *FUS* mRNA levels remained comparable across the three genotypes with hFUS transgene (Fig. 4 C and Fig. S2).

**Fig. 5:**
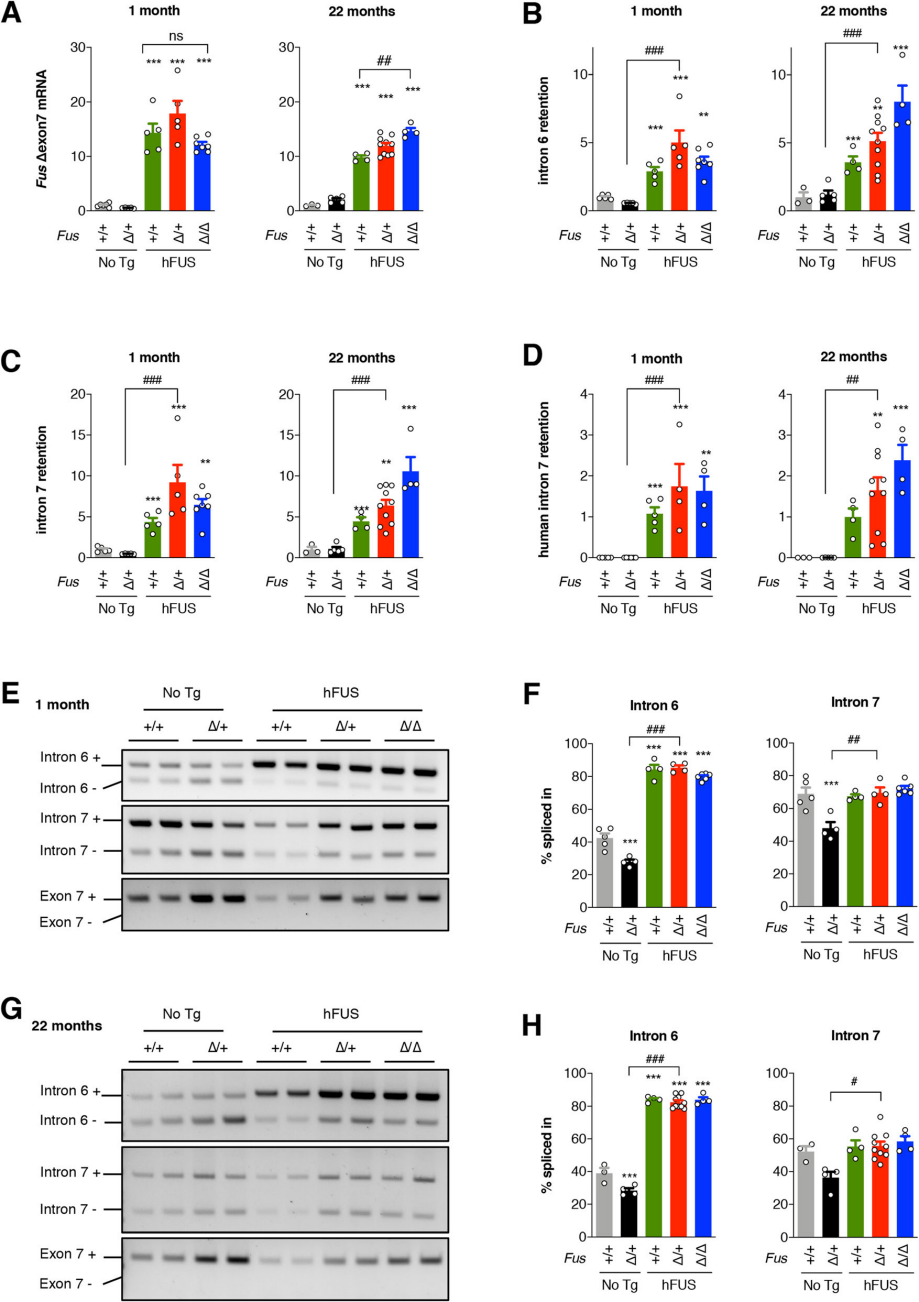
hFUS transgene activates autoregulatory splicing in *Fus*^∆NLS/+^ spinal cord. **A-D**: RT-qPCR results for endogenous *Fus* mRNA deleted of exon 7 (**A**), endogenous *Fus* mRNA retaining intron 6 (**B**), endogenous *Fus* mRNA retaining intron 7 (**C**) and exogenous *FUS* mRNA retaining intron 7 (**D**) in spinal cord at 1 month (left) or 22 months (right) of age. Note that the hFUS transgene activates autoregulatory exon 7 skipping as well as retentions of introns 6 and 7 in endogenous mRNA and retention of intron 7 in exogenous mRNA at 1 and 22 months of age. *N* = 4–8. *, p < 0.05, **p < 0.01, ***p < 0.001 vs. *Fus*^*+/+*^, #, *p* < 0.05, ###, *p* < 0.001 vs. indicated genotype by ANOVA followed by Tukey. **E-H**: representative gel electrophoresis of RT-PCR assays identifying RNA species with or without intron 6 retention (**E**, **G**, upper panel), with or without intron 7 retention (**E**, **G**, middle panel), or with or without exon 7 skipping (**E**, **G**, lower panel) in spinal cord at 1(**E**) or 22 (**G**) months of age. We did not detect exon 7 skipped mRNA using these assays. Panels F and H show the percentage of intron 6 or 7 retention (intron + band intensity divided by the sum of intensities of intron + and intron – bands, multiplied by 100), for 1 month (**F**) or 22 months (**H**) of age. *N* = 4–8. ***p < 0.001 vs. *Fus*^*+/+*^, #, *p* < 0.05, ##, *p* < 0.01, ###, *p* < 0.001 vs. indicated genotype by ANOVA followed by Tukey

We further investigated the three possible autoregulatory mechanisms that have been documented for FUS (Fig. S3). First, FUS protein is proposed to bind to its own pre-mRNA, leading to the splicing of exon 7, and the possible subsequent degradation of the abnormally ∆exon 7 *FUS* mRNA through nonsense-mediated mRNA decay [15, 20]. Interestingly, expression of hFUS transgene increased levels of the aberrantly spliced *Fus* ∆exon 7 mRNA (Fig. 5 A and Fig. S4A). Secondly, increased FUS levels have recently been reported to lead to the retention of introns 6 and 7 in the mature mRNA, and to the nuclear retention of the aberrant transcripts [14]. *Fus* endogenous mRNAs with retained introns 6 or 7 strongly increased in all mice expressing hFUS transgene at 1- and 22-months of age (Fig. 5B-C and Fig. S4B-C). We also observed prominent retention of human intron 7 in all samples derived from mice expressing the hFUS transgene (Fig. 5D and Fig S4D), which is consistent with the strong conservation of introns 6 and 7 between species (Fig S5). Thirdly, besides intron skipping and retention, FUS has also been reported to regulate its own levels through the stimulation of miR200 [16]. Another target of miR200 is ZEB1, whose expression is dependent upon levels of miR200 [21, 22]. Here, *Zeb1* expression appears unchanged in *Fus*^∆NLS/+^ tissues, whether or not expressing the h*FUS* transgene (Fig. S6), indirectly suggesting that this latter autoregulatory mechanism is not engaged in the effects mediated by the hFUS transgene.

To identify the predominant autoregulatory mechanism(s) contributing to reduction of mutant FUS by hFUS, i.e. intron retention and/or exon skipping, we performed RT-PCR using trios of oligonucleotides allowing to calculate a percentage of intron retention or exon skipping relative to the total amount of *Fus* mRNA. As shown in Fig. 5E, and quantitated in Fig. 5 F and H (uncropped gels shown in Source data), a significant proportion of mouse *Fus* mRNA retained either intron 6 or intron 7 in wild-type tissues, and this proportion decreased with the ∆NLS transgene, consistent with inhibition of autoregulatory intron retention in these conditions [14]. Conversely, the large majority of mouse *Fus* mRNA appeared to retain intron 6 or intron 7 in the presence of hFUS transgene (Fig. 5E-H, Fig. S4). In the same conditions, we were unable to detect a significant proportion of ∆exon 7 mouse mRNA, suggesting that, while this species can be detected using RT-qPCR (Fig. 5E**)**, it is quantitatively minor. Collectively, these data indicate that wild-type human FUS gene decreases expression of the endogenous *Fus* gene through increased retention of introns 6 and 7 leading to decreased production of toxic FUS^∆NLS^ protein, and subsequent alleviation of all the downstream consequences of the expression of cytoplasmically mislocalized mutant FUS.

## Discussion

In the current study, we show that providing a wild-type allele of the *FUS* gene is sufficient to rescue ALS-like phenotypes associated with cytoplasmically retained mutant FUS protein expression. Our result appears *a priori* paradoxical since the toxicity of FUS mutations was shown to be largely driven by cytoplasmic FUS [7–12], that is not expected to be directly compensated by the wild-type protein. Furthermore, overexpression of the wild-type protein was shown to be toxic to neurons [9, 17, 23].

### The wild-type FUS transgene rescues phenotypes associated with the ***Fus***^***∆NLS***^ mutation

In this study, the hFUS transgene displayed broad protective effects against the lethality driven by the *Fus*^*∆NLS*^ allele in homozygosity. We previously showed that *Fus*^*∆NLS/∆NLS*^ mice die at birth due to an inability to inflate lungs [11]. Here, this perinatal lethality was fully prevented by the hFUS transgene, and numbers of *Fus*^*∆NLS/∆NLS*^ mice expressing the hFUS transgene were obtained at the expected mendelian ratio, consistent with a full rescue. However, and in spite of surviving the perinatal period, a proportion of homozygous rescued mice died prematurely and abruptly at an adult age (about 20 % by one year of age). The only noticeable phenotype observed in these mice was an age-related decrease in body weight gain, but we did not observe prominent weakness, nor obvious ALS-related symptoms. A possible cause of death could be an increased sensitivity to epileptic seizures, as we recently showed that *Fus*^*∆NLS/+*^ mice display increased spontaneous cortical neuronal activity [18]. Further work on these mice is required to finely characterize their phenotypes and pinpoint to the cause of their premature death.

How can the hFUS transgene rescue the perinatal lethality of *Fus*^*∆NLS/∆NLS*^ mice? Perinatal death of *Fus*^*∆NLS/∆NLS*^ mice is similar to that of mice with a complete ablation of FUS. This suggests that the presence of FUS in the nucleus during development is required to bypass the perinatal period. Thus, it is likely that the hFUS transgene allows for the production of sufficient functional nuclear FUS to overcome the perinatal lethality of *Fus*^*∆NLS/∆NLS*^ mice. Unexpectedly, a significant fraction of the mouse FUS protein was found in the nucleus of adult *Fus*^*∆NLS/∆NLS*^ mice with hFUS transgene, albeit the endogenous FUS protein is completely truncated of the NLS. This is *a priori* surprising as the NLS is the major domain responsible for interaction with nuclear import receptors such as karyopherin ß2 [24, 25]. There are at least two possible explanations to this observation. First, the human FUS protein could support the nuclear import of the mutant ∆NLS mouse protein as wild-type and mutant FUS interact with each other [26]. Second, nuclear import of FUS might be possible through NLS-independent mechanisms. Indeed, recent work has shown that ∆NLS mutants of FUS could still interact through RGG domains with karyopherin ß2 [24, 25], as well as other nuclear import receptors [25] leading to significant nuclear import [24] .

Consistent with the protection offered in homozygous mice, the hFUS transgene prevented premature death and muscle weakness in *Fus*^*∆NLS/+*^ mice. Importantly, the hFUS transgene had no effect *per se* on survival of wild-type mice. About 30 % of *Fus*^*∆NLS/+*^ mice died before 2 years of age, which is consistent with previous findings reported in another knock-in model of *FUS-*ALS [10]. Further confirming the protection offered by the hFUS transgene, the motor defect of *Fus*^*∆NLS/+*^ mice was also rescued. It should be noted however that the expression of the transgene led to mild motor defects, mostly in males in one of the tests (grip strength) used. This suggests that the protection offered by the hFUS transgene might be accompanied with toxicity appearing with age, echoing a recent report on the toxicity of viral overexpression of SMN in spinal muscular atrophy mice [27]. In all, our results demonstrate broad protective effects of the hFUS transgene on the deleterious phenotypes associated with either homozygous or heterozygous *Fus*^*∆NLS*^ mutation, yet not excluding some residual toxicity associated with the transgene expression.

### The wild-type hFUS transgene mitigates disease through autoregulatory mechanisms

Importantly our work demonstrates that the wild-type transgene activates FUS autoregulatory loop to mitigate the phenotype. A first possible protective mechanism could have been that the hFUS transgene rescues a loss of nuclear FUS. However, we did not observe any loss of nuclear FUS in *Fus*^*∆NLS/+*^ mice. Alternatively, the hFUS transgene appears to indirectly protect from accumulation of mutant protein through the autoregulatory loop maintaining nuclear FUS levels [14, 15, 20] to avoid the toxicity of loss of nuclear FUS [28–32] or its excess [9, 17, 23]. We provide several lines of evidence demonstrating the engagement of FUS autoregulation upon expression of the hFUS transgene. First, *Fus* mRNA and protein levels are increased in *Fus*^*∆NLS/+*^ mice, thereby compensating the proportion of FUS protein translated from the mutant allele and unable to enter the nucleus. Conversely, in single hFUS transgenic mice the addition of the exogenous *FUS* transgene is sufficient to decrease endogenous mouse *Fus* mRNA levels, consistent with previous studies [8, 9]. Here, the addition of the hFUS transgene in *Fus*^*∆NLS/+*^ mice rescued overexpression of endogenous *Fus* in *Fus*^*∆NLS/+*^ mice, and decreased mutant mRNA levels. Since this overexpression acts as a feed forward mechanism amplifying the cytoplasmic accumulation of FUS, avoiding this overexpression might on its own be sufficient to slow down the vicious cycle leading to phenotypes in *Fus*^*∆NLS/+*^ mice. Consistently, our RT-PCR experiments demonstrate that a significant proportion of *Fus* mRNA retains introns 6 and 7, and that the percentages of intron retention in the endogenous *Fus* mRNA increase strikingly with the expression of the hFUS transgene. Importantly, the exogenous human transgene is also, on its own, subject to autoregulation in *Fus*^*∆NLS/+*^ mice, despite the heterologous system. This is consistent with the strikingly high conservation of introns 6 and 7 of the *FUS* gene between species suggesting that autoregulation of *FUS* is critical for its functions (Fig. S5). Of note, the existence of autoregulation of the human transgene in the mouse model is a plausible explanation for the high toxicity of cDNA-based constructs devoid of required autoregulatory sequences, and the relative innocuity of genomic based constructs [8, 9, 17].

Our findings are in agreement with previous studies identifying retention of introns 6 and/or 7 as the major mechanism of *Fus* autoregulation [14]. Indeed, about half of the endogenous *Fus* transcript appears retaining either intron 6 or 7 in the cortex or spinal cord of wild-type mice. Consistent with the results of Humphrey and collaborators, the ∆NLS mutation leads to decreased retention of these two introns in both tissues. On the contrary, the introduction of the hFUS transgene leads to substantial retention of both introns, with nearly 90 % of endogenous *Fus* mRNA having intron 6 retained. The effect of the hFUS transgene appeared less marked on retention of intron 7, albeit this intron carries most of the FUS binding sites on the pre-mRNA [14, 15, 20]. In addition to the substantially increased intron retention upon hFUS transgene expression, we also observed enhanced exon 7 skipping. This mRNA species appeared however minor, as it was not observed using splicing assays, and required 4–6 supplementary PCR cycles to be detectable. While this suggests that exon skipping is a minor mechanism of FUS autoregulation in our *in vivo* model, our current results do not allow to completely exclude its contribution as Zhou and collaborators demonstrated that ∆exon 7 *Fus* mRNA is subject to non-sense mediated mRNA decay (NMD) [15]. Nevertheless, other studies suggest that NMD is not involved in FUS autoregulation, at least in cultured cells [14]. Further, our study did not find evidence of altered miR141/200/ZEB1 pathway [16] by measuring *Zeb1* mRNA levels. Thus additional work is needed to fully evaluate the contribution of this pathway to *in vivo* FUS autoregulation. In all, our efforts are consistent with a predominant role of intron retention in FUS autoregulation *in vivo*, and further research is warranted to identify which intron is critical for this process.

### Possible consequences for therapeutic strategies in ***FUS***-ALS

Our results suggest that gene therapy to reintroduce the wild-type protein, while including sequences required for autoregulation, would enable the correction of molecular and behavioral phenotypes, meanwhile avoiding the toxicity of wild-type protein overexpression in *FUS-*ALS. Our work provides a proof of concept for a potential therapeutic strategy, albeit there are limitations to overcome before clinical translation. First, our study has been obtained in a heterologous system, with a human *FUS* transgene expressed in mouse cells. While the very high conservation of intronic sequences (Fig S5) gives hopes that a similar intervention should be protective also in human cells, an intermediate validation step using a human cell model is warranted. Second, our current results have been obtained using a complete *FUS* gene inserted through classical transgenesis in a locus independent of the mouse *Fus* gene. To translate these results in a therapeutically viable strategy, it would be first necessary to use a gene therapy vector, such as an adeno-associated virus (AAV), to provide the equivalent of our hFUS transgene. Thus, an important effort of sequence optimization is required to shorten the lead “therapeutic” construct, in order to allow introduction of a potential therapeutic sequence into the viral vector. Future research should thus aim at identifying the minimal sequence requirements for FUS autoregulation to ultimately engineer a small autoregulation competent expression construct.

Besides *FUS*-ALS, FUS mutations have been associated with other neurodegenerative diseases, such as frontotemporal dementia [33–35], chorea [36], mental retardation [37], psychosis [38] and essential tremor [39]. FUS aggregation has been observed in sporadic ALS [40–42] and FTD [43–45], but also in spino-cerebellar ataxia and Huntington’s disease [46, 47]. A gene therapy to restore normal nuclear FUS levels might thus be relevant for other patients to be identified. Last, it is noteworthy that similar autoregulatory mechanisms exist for other RNA-binding proteins, in particular TDP-43 [48–53] or hnRNPA1 [54]. Whether utilizing such autoregulatory mechanisms to decrease mutant protein through overexpression of a wild-type protein might be a general therapeutic approach in such diseases remains to be determined.

## Conclusions

Our results show that the phenotypes triggered by a cytoplasmically retained FUS protein associated to ALS can be rescued by a wild type *FUS* allele. The wild-type *FUS* allele activates the homeostatic autoregulatory loop triggering retention of introns 6 and 7 in the endogenous *Fus* mRNA, leading to decreased mutant protein load. Our work provides a proof of concept for a potential gene therapy strategy for FUS-ALS.

## Materials and methods

### Mouse models and genotyping

Mouse experiments were approved by local ethical committee from Strasbourg University (CREMEAS) under reference number 2,016,111,716,439,395 and all experimental procedures performed in San Diego were approved by the Institutional Animal Care and Use Committee of the University of California, San Diego. Transgenic mice were generated as described in [11, 12] and [8], were bred in Charles River animal facility and housed in the Faculty of medicine from Strasbourg University with 12/12 hours of light/dark cycle (light on at 7:00 am) under constant conditions (21 ± 1 °C; 60 % humidity) and with unrestricted access to food and water.

Mice were weaned and genotyped at 21 days by PCR from tail biopsy, or at death if occurring before 21 days of age.

The following primer sequences were used to genotype mice:

hFUS-For: GAATTCGTGGACCAGGAAGGTC.

hFUS-Rev: CACGTGTGAACTCACCGGAGTCA.

FUS-For: GAT-TTG-AAG-TGG-GTA-GAT-AGT-GCA-GG.

FUS-Rev: CCT-TTC-CAC-ACT-TTA-GTT-TAG-TCA-CAG.

Heterozygous *Fus*^∆NLS/+^ knock-in mice, lacking the PY-NLS, were crossed with mice expressing human wild type FUS from a complete, autoregulatory competent, human gene to obtain following genotypes: *Fus*^+/+^, *Fus*^*ΔNLS*/+,^*Fus*^*ΔNLS*/ΔNLS^, *Fus*^+/+^/hFUS, *Fus*^∆NLS/+^/hFUS, *Fus*^ΔNLS/ΔNLS^/hFUS. The genetic background of all mice used in this study is C57Bl6/J. Breeding steps were performed in parallel in both laboratories. 76 mice of the F2 generation were generated in Strasbourg, and 110 mice of the F2 generation were generated in San Diego.

### Mouse behavior

#### Survival

Survival was studied during the first hours after birth and dead new born mice were genotyped. Mice surviving the post-natal period were genotyped at 21 days and followed weekly until death or euthanized using ketamine-xylazine when they reach the following endpoints: auto-mutilation, weight loss greater than 10 % of the initial weight and when they could not turn around again within 10 s after being laid on their side.

#### Inverted grid

Mice were habituated for 30 min in the test room prior testing. Motor performance was assessed weekly as described previously [12] from 1 month until 22 months of age. The wire grid hanging time (or “hang time”) was defined as the amount of time that it takes the mouse to fall down from the inverted grid and was measured visually with a stopwatch. The procedure was repeated 3 times during 5 min with 5 min break between tests. All mice were returned to their homecage after completing the test. The holding impulse corresponds to hanging time normalized with mouse weight and gravitational force.

#### Grip test

Grip strength was measured using a Grip Strength Meter (Columbus Instruments, Columbus, OH) on cohorts (N = 12–30) made up of approximately the same number of males and females. Mice were allowed to grip a triangular bar only with hind limbs, followed by pulling the mice until they released; five force measurements were recorded in each separate trial.

### Histological techniques

Mice aged of 22 months were anesthetized with intraperitoneal injection of 100 mg/kg ketamine chlorhydrate and 5 mg/kg xylazine then perfused with PFA 4 %. After dissection, spinal cord was included in agar 4 % and serial cuts of 40 μm thick were made with vibratome.

#### Peroxidase immunohistochemistry

For peroxidase immunohistochemistry, sections were incubated 10 min with H_2_O_2_ 3 %, washed 3 times and blocked with 8 % Horse serum, 0,3 % Bovine Serum Albumin and 0,3 % Triton in PBS with 0,02 % Thimerosal. Sections were incubated with rabbit anti-FUS antibody (ProteinTech 11570-1-AP; diluted 1:100) in blocking solution overnight at room temperature. After washing sections, they were incubated for 2 h at room temperature with biotinylated donkey anti-rabbit antibody (Jackson 711-067-003; diluted 1:500) in blocking solution. Then, sections were washed, incubated for 1 h in horseradish peroxidase ABC kit (Vectastain ABC kit, PK-6100, Vector Laboratories Inc.), washed and incubated with DAB (Sigma, D5905). The enzymatic reaction was stopped by adding PBS 1X and washed with water. Finally, sections were mounted with DPX mounting medium (Sigma, O6522).

#### Immunofluorescence

After epitope retrieval in 10 mM citrate pH6.0 30 min at 80 °C, sections were incubated in blocking solution (5 % Horse serum, 1 % Triton in PBS) at room temperature for 30 min, then incubated overnight at room temperature in primary antibody in PBS + 0.1 % triton X100: rabbit anti-FUS antibody (total FUS) (ProteinTech, 11570-1-AP, 1:100), Rabbit anti-C-ter FUS (Bethyl, A300-294 A, 1/100), Rabbit anti-mouse FUS[8], goat anti-ChAT (Millipore, AB144P, 1/50), rat anti-ADMA FUS ([5, 6], kind gift of Pr C. Haass, Munich Germany, 1/20). After 3 rinses in PBS, sections were incubated for 2 h at room temperature with Hoechst (Sigma, B2261, 1/50.000) and secondary antibodies in blocking solution: Alexa-488-conjugated donkey anti-rabbit secondary antibody (Jackson, 711-547-003, 1/500) Alexa-488-conjugated donkey anti-rat secondary antibody (Jackson 712-545-153 1/1000) or Alexa-594-conjugated donkey anti-goat secondary antibody (Molecular Probes, A 11,058, 1/500). Finally, sections were subsequently washed with PBS 1 × (3 x 10 min) and mounted in Aqua/polymount (Polysciences 18,606).

Immunofluorescence staining was monitored with a laser scanning microscope (confocal LSM 800 Zeiss) equipped with 40 × oil objective (NA1.4). Excitation rays are sequential argon laser 488nm, diode 561nm, diode 405nm. Emission bandwidths are 500-570nm for Alexa488, 570-617nm for Alexa594, and 400-500nm for Hoechst. Single-layer images were analyzed using ImageJ freeware (http://rsbweb.nih.gov/ij/).

### Tissue homogenization, fractionation and western blotting

Total protein extracts were obtained from brain homogenization using zirconium oxide beads (Bertin Technologies) in combination with Precellys Tissue homogenizer (Bertin Technologies) for 3 × 15 s, 5000 rpm in RIPA buffer (Tris-HCl pH 8 50mM, sodium chloride 150mM, sodium deoxycholate 0.5 %, SDS 0.1 %, Triton-X100 1 %). The supernatants were collected after centrifugation for 15 min, 14,000 rpm at 4 °C and the protein extracts were measured with Pierce™ BCA Protein Assay Kit (Thermo Scientific). SDS-PAGE was performed with 10 µg of total protein extracts using Mini-PROTEAN TGX gel 4–15 % (Biorad). Proteins were blotted on PVDF membrane using Mini Trans-Blot® Cell (Biorad) and blocked with 10 % non-fat milk during 1 h. Primary antibodies (Rabbit anti-hFUS (1/2000), Rabbit anti-mFUS (1/4000), Rabbit anti-FUS (total FUS) (Bethyl, A-300-293 A, 1/2000), Rabbit anti-C-ter FUS (Bethyl, A300-294 A, 1/2000), Mouse-anti-vinculin (Merk Millipore, V9131, 1/2000)) were incubated overnight at 4 °C in 3 % non-fat milk. Washing was proceeded with washing buffer (Tris pH 7.4 1 M, NaCl 5 M, Tween 20 0.1 %) and secondary antibodies (anti-rabbit HRP (Agilent, P0448, 1/5000), anti-mouse HRP (Jackson Immunoresearch, 715-035-150, 1/5000) were incubated 1h30 at room temperature. After successive washes, proteins were visualized with chemiluminescence using SuperSignal™ West Pico PLUS Chemiluminescent Substrate (Thermo Scientific, 34,577) and chemiluminescence detector.

Tissues were washed in PBS1x and lysed in NE-PER Nuclear and Cytoplasmic Extraction (Thermo Scientific, 78,835) according to the manufacturer’s instructions. Protein extracts were dosed by BCA Assay (Interchim, UP95424A, UP95425A). Thereafter proteins were denatured and SDS page was performed with 30 µg of cytoplasmic proteins and 10 µg of nuclear proteins on criterion TGX stain free gel 4–20 % (Biorad, 5,678,094). Proteins were blotted on nitrocellulose membrane using semi-dry Transblot Turbo system (BioRad, France) and blocked with 10 % non-fat milk during 1 h. Primary antibodies (Rabbit anti-hFUS ([8, 9], #14,080, 1/2000), Rabbit anti-mFUS ([8, 9], #14,082, 1/4000), Rat anti-FUS ADMA ([5, 6], kind gift of Pr C. Haass, Munich Germany, 1/500), Rabbit anti-FUS (total FUS) (Bethyl, A-300-293 A, 1/2000), Rabbit anti-C-ter FUS (Bethyl, A300-294 A, 1/2000), Sheep anti-SOD1 (Calbiochem, 574,597, 1/1000), Rabbit anti-HDAC1 (Bethyl, A300-713 A, 1/1000) ) were incubated overnight at 4 °C in 3 % non-fat milk. Washing was proceeded with washing buffer (Tris pH 7.4 1 M, NaCl 5 M, Tween 20 100 %) and secondary antibodies (anti-rabbit HRP (PARIS, BI2407,1/5000), anti-sheep HRP (Jackson, 713-035-147, 1/5000)) were incubated 1h30 at room temperature. After successive washes, proteins were visualized with chemiluminescence using ECL Lumina Forte (Millipore, France) and chemiluminescence detector (Bio-Rad, France). Total proteins were detected with stain free gel capacity (Biorad, 5,678,094) and used to normalize for protein loading. All values were normalized against nuclear levels of FUS in *Fus*^+/+^ extracts set to 1.

### RNA extraction and RT-qPCR

Total RNA was extracted from spinal cord and frontal cortex using TRIzol® reagent (Life Technologies). 1 µg of RNA was reverse transcribed with iScript™ reverse transcription (Biorad, 1,708,841). Quantitative polymerase chain reaction was performed using Sso Advanced Universal SYBR Green Supermix (Bio-Rad 1,725,274) and quantified with Bio-Rad software. Gene expression was normalized by calculating a normalization factor using actin, TBP and pol2 genes according to GeNorm software [55].

Primer sequences are provided in Table S1.

### RT-PCR

1 µg of RNA was reverse transcribed with iScript™ reverse transcription (Biorad, 1,708,841). Polymerase chain reaction was performed using in 25 µL microtubes with MasterMix Taq DNApolymerase (VWR International, Ref. 733–1320) using the following programs: Intron 6 retention and exon 7 skipping (5 min 95 °C, (30 s 95 °C, 30 s 56 °C, 30 s 68 °C)x 30; 5 min 68 °C), Intron 7 retention (5 min 95 °C, (30 s 95 °C, 30 s 61 °C, 30 s 68 °C)x 30; 5 min 68 °C), 10 µL of the PCR products were loaded on a 2 % agarose (Euromedex, Ref.D5-E) gel electrophoresis with Low Molecular Weight DNA Ladder (NEB, Ref. N3233L) and stained with ethidium bromide using standard procedures. For quantification, we quantified individually the signal intensities of the two bands, and computed a % of intron retention as such: (intensity of Intron + band )/ (intensity of Intron + band + intensity of Intron- band)*100. We did not quantify a percentage of exon 7 skipping as the exon 7 skipped product was below the detection threshold of the assay.

### Statistics

All results from analysis are presented as mean ± standard error of the mean (SEM) and differences were considered significant when p < 0.05. Significance is presented as follows: * *p* < 0.05, ** *p* < 0.01, and *** *p* < 0.001. For comparison of two groups, two-tailed unpaired Student’s t –test was used in combination with F-test to confirm that the variances between groups were not significantly different. For longitudinal analysis of behavioral data, results were analyzed using a mixed effect analysis with three factors (∆NLS genotype, hFUS genotype and age) as indicated in the figure legends. Data were analyzed by using the GraphPad Prism version 8.0.

## Supplementary Information

**Additional file 1.**

**Additional file 2.**

**Additional file 3.**

## Data Availability

Data and material are available upon reasonable request to corresponding authors;

## Abbreviations

ADMA: Asymmetrically dimethylated arginine
ALS: Amyotrophic lateral sclerosis
BAC: Bacterial artificial chromosome
CHAT: Choline acetyl transferase
FTD: Fronto-temporal dementia
FUS: Fused in sarcoma
hnRNPA1: Heterogeneous ribonucleoprotein particle A1
NLS: Nuclear localization sequence
TDP-43: TAR DNA binding protein of 43 kDa
