# Supplementary material for: Wild-type FUS corrects ALS-like disease induced by cytoplasmic mutant FUS through autoregulation
Source: Mol Neurodegener. 2021 Sep 6;16:61. doi: 10.1186/s13024-021-00477-w (PMC8419956; doi:10.1186/s13024-021-00477-w)

Sanjuan-Ruiz et al. Source data

Uncropped western blots Figure 2A

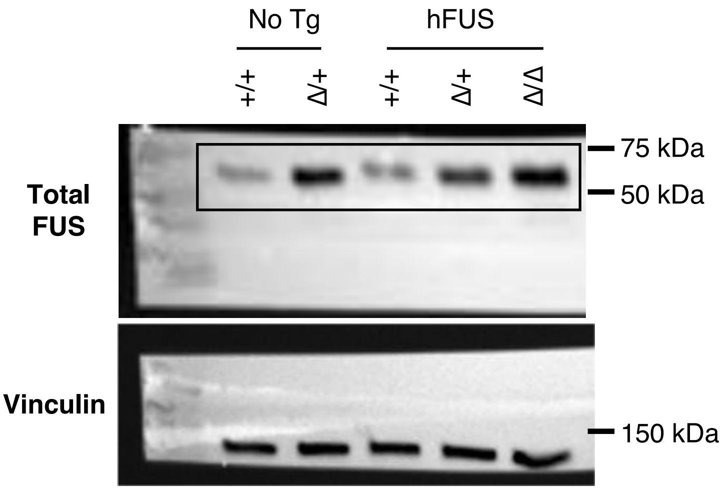

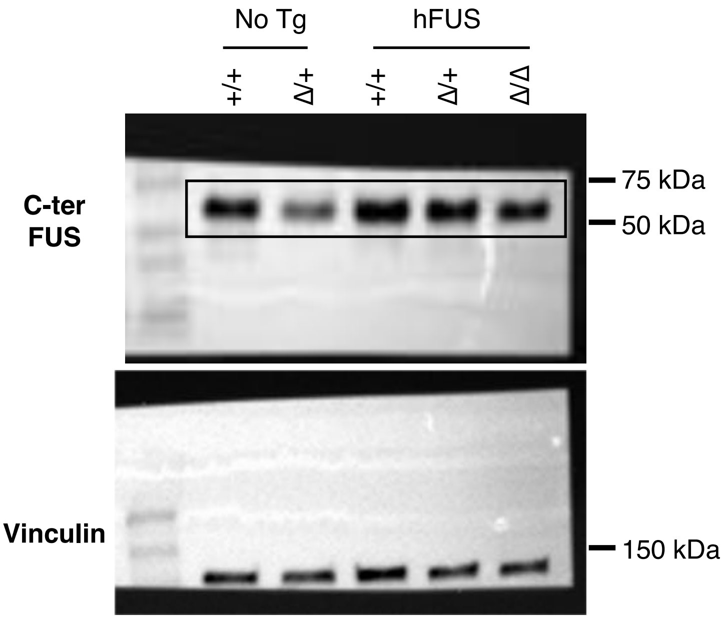

Sanjuan-Ruiz et al. Source data

Uncropped western blots Figure 2A (continued)

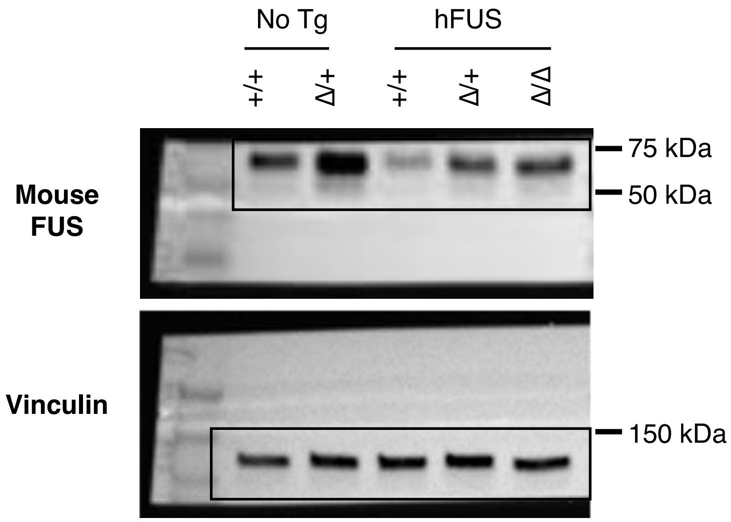

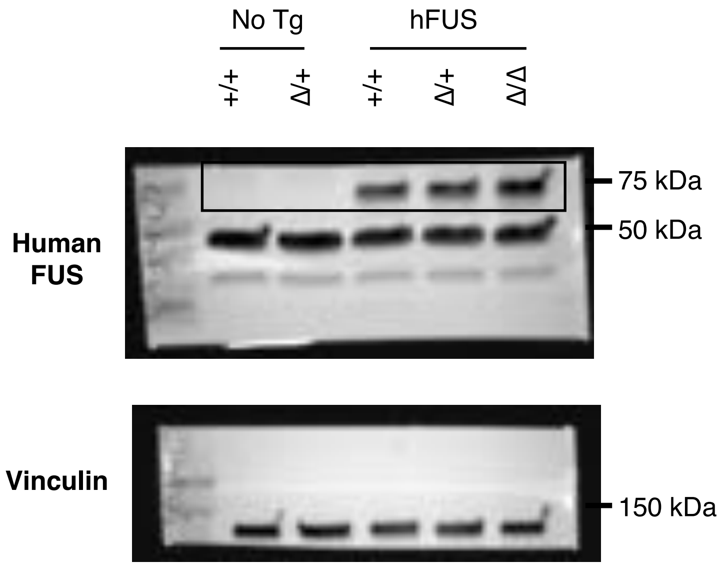

Sanjuan-Ruiz et al. Source data

__Uncropped western blots Figure 2C__

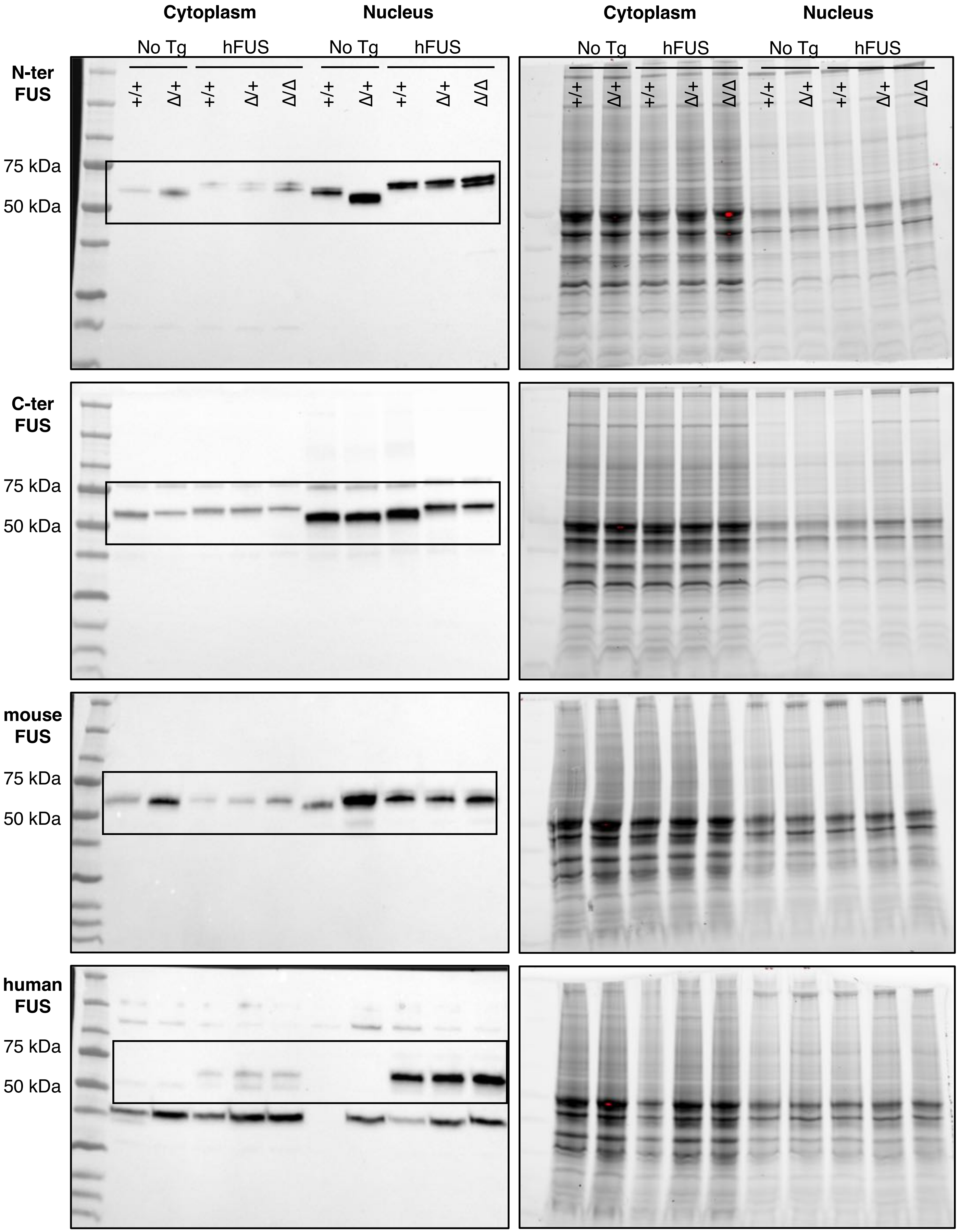

Sanjuan-Ruiz et al. Source data

Uncropped western blots Figure 2C (continued)

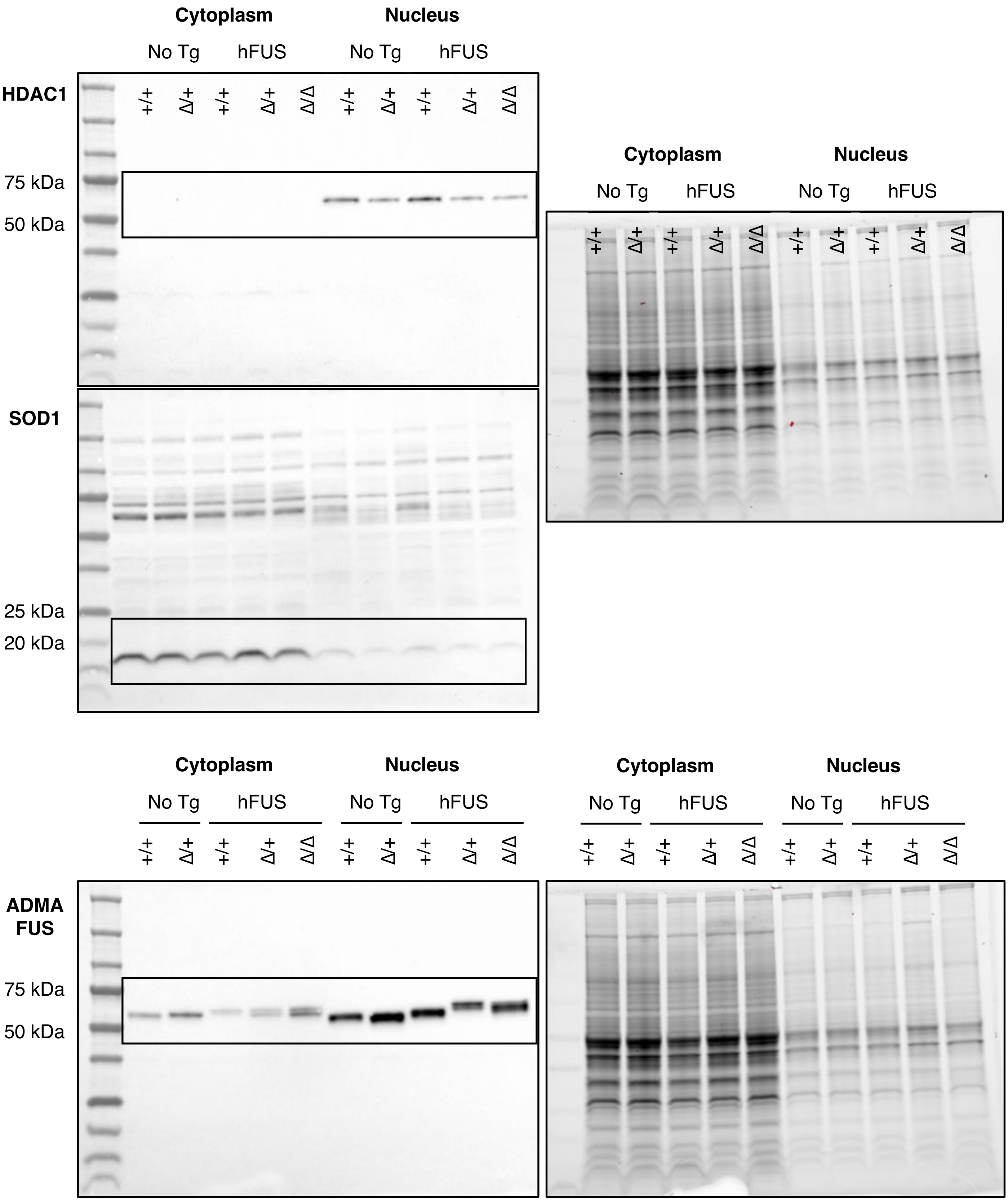

Sanjuan-Ruiz et al. Source data

Uncropped gels

Figure 5

Intron 6 retention

Lumbar spinal cord
1 month

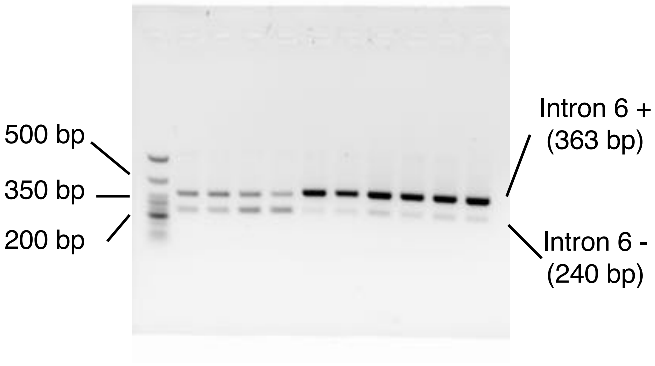

Lumbar spinal cord
22 months

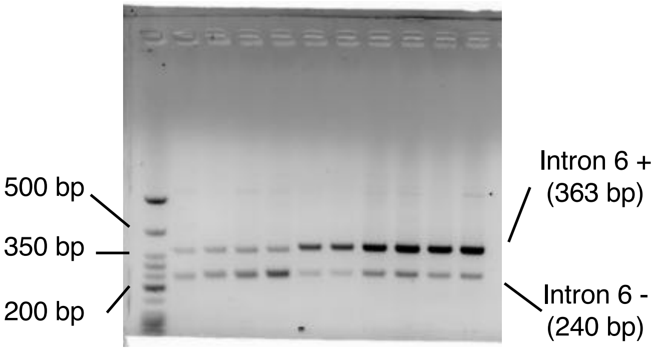

Intron 7 retention

Lumbar spinal cord
1 month

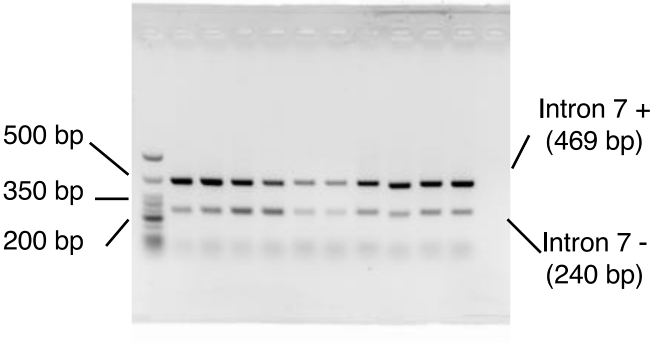

Lumbar spinal cord
22 months

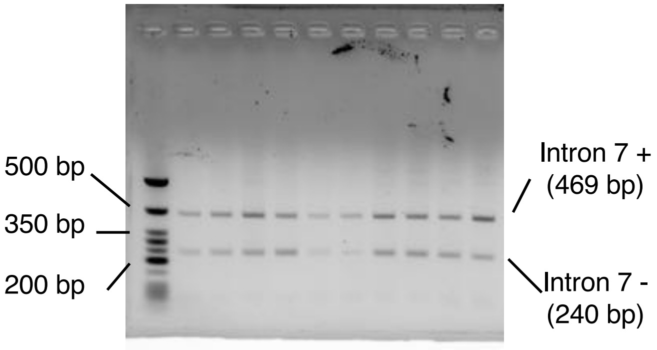

Intron 7 skipping

Lumbar spinal cord
1 month

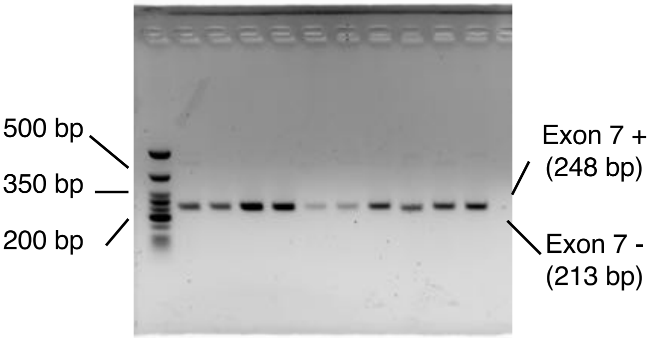

Lumbar spinal cord
22 months

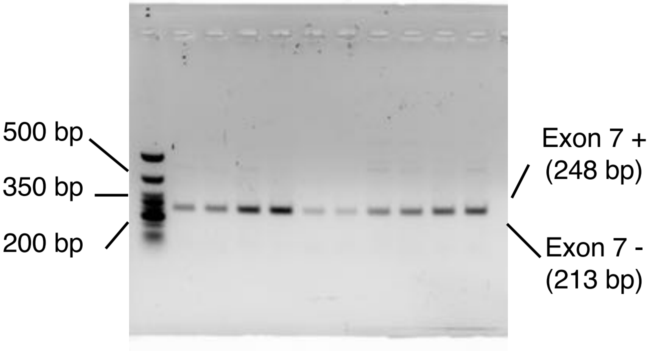

Sanjuan-Ruiz et al. Source data

<u>**Uncropped gels**</u>

Figure S5

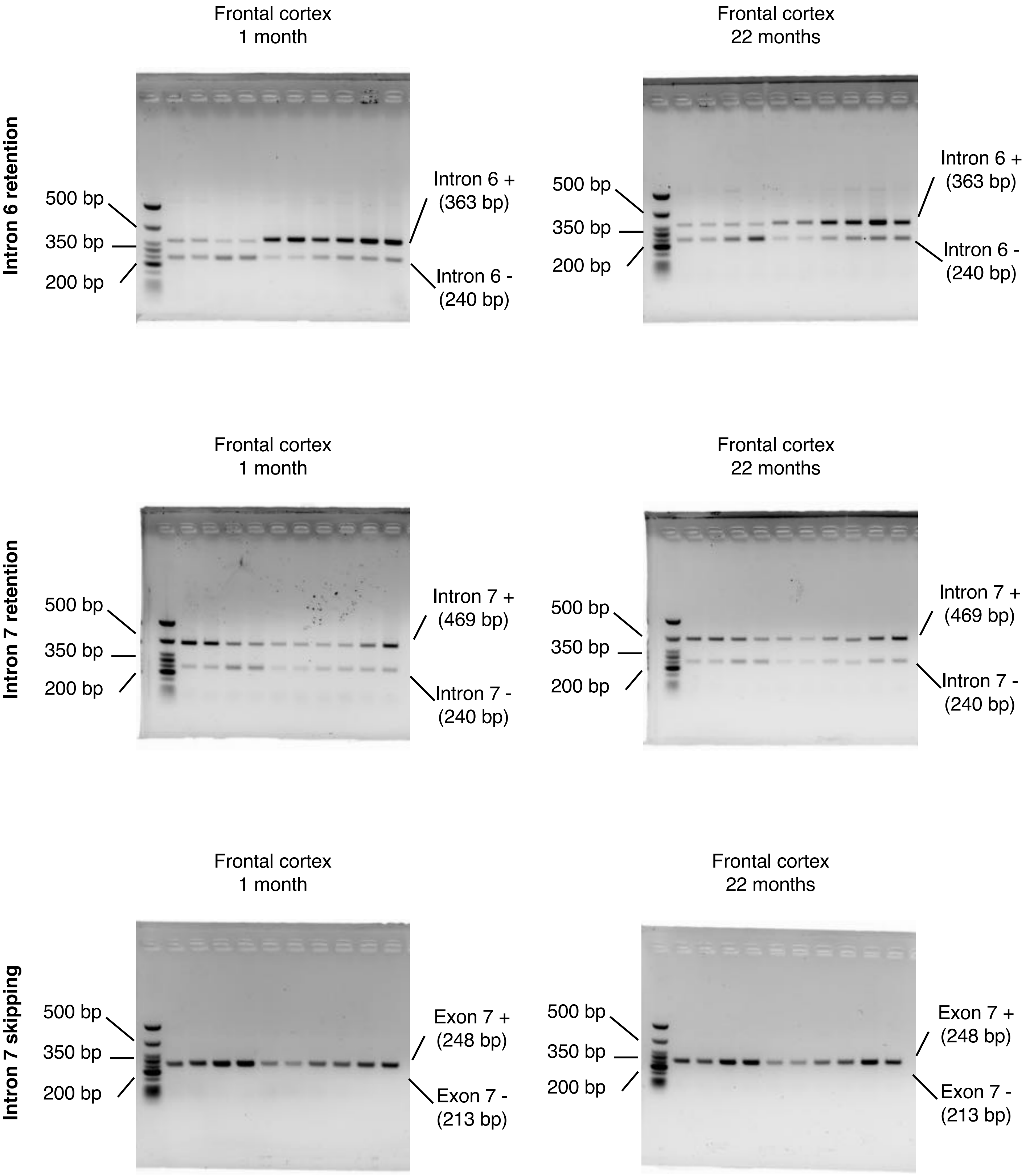

Supplement: Supplementary file 1 — Additional file 1. [file 13024_2021_477_MOESM1_ESM.pdf]
