# Supplementary material for: Wild-type FUS corrects ALS-like disease induced by cytoplasmic mutant FUS through autoregulation
Source: Mol Neurodegener. 2021 Sep 6;16:61. doi: 10.1186/s13024-021-00477-w (PMC8419956; doi:10.1186/s13024-021-00477-w)
Supplement: Supplementary file 2 — Additional file 2. [file 13024_2021_477_MOESM2_ESM.pdf]

Wild-type FUS corrects ALS-like disease induced by cytoplasmic mutant FUS through autoregulation.

SANJUAN-RUIZ *et al.*

Supplementary Information

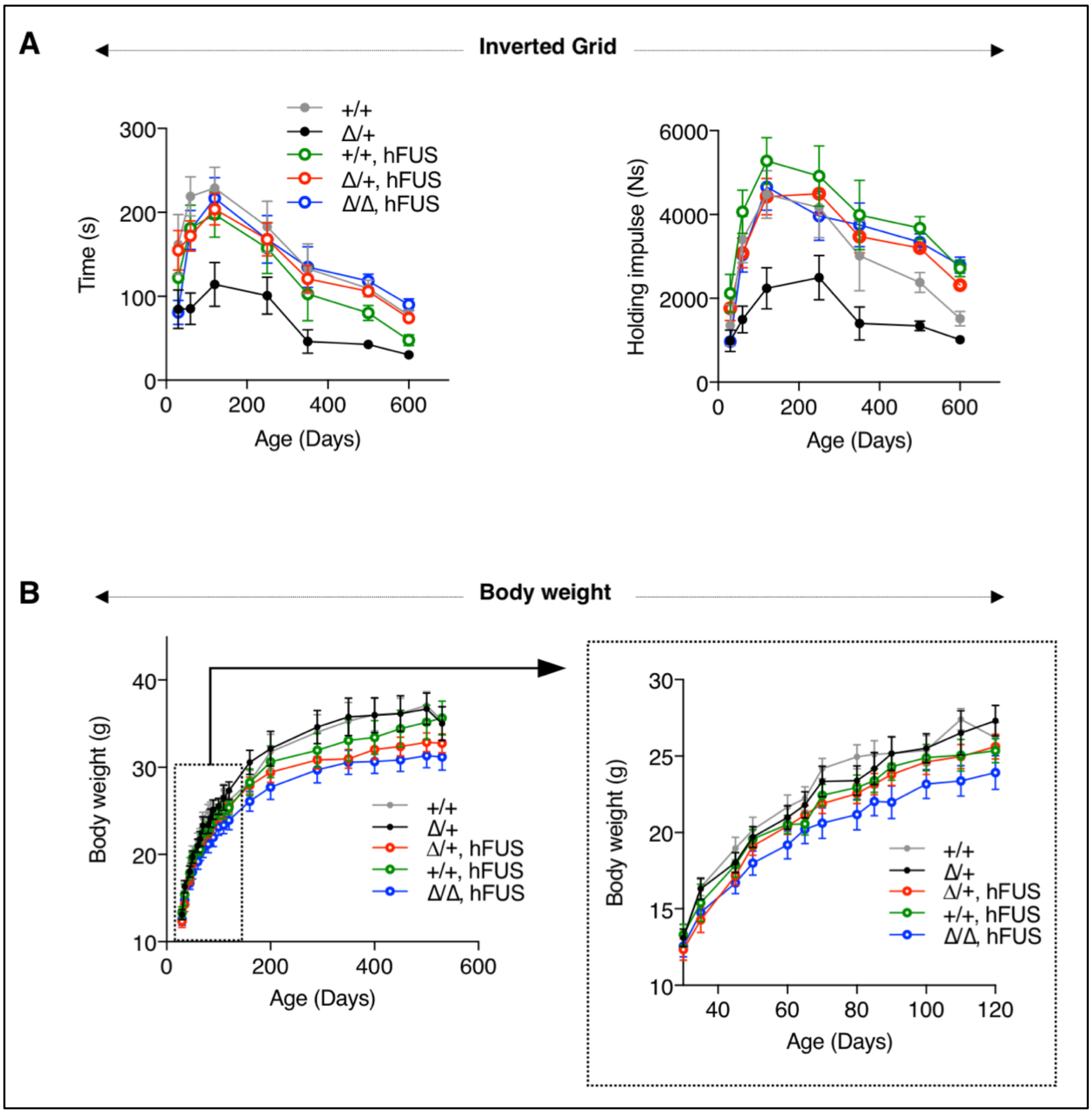

Supplementary figure 1: additional behavioral characterization
A: Age-dependent changes in the mean hanging time (s) and holding impulse (Ns) in the four-limb wire inverted grid test in *Fus*$^{+/+}$, *Fus* $^{+/+}$, and *Fus*$^{\Delta NLS/+}$ mice with or without hFUS transgene and *Fus*$^{\Delta NLS/\Delta NLS}$ mice with hFUS transgene.
Experimental data for the control group are identical to Figure 1, as mice were littermates and followed simultaneously.
B: Body weight of *Fus*$^{+/+}$ (+/+), *Fus*$^{\Delta NLS/+}$ mice (Δ/+-) with or without hFUS transgene and *Fus*$^{\Delta NLS/\Delta NLS}$ mice (Δ/Δ) with hFUS transgene. Data are derived from experiments presented in Figure 1, with balanced sex ratio between groups.

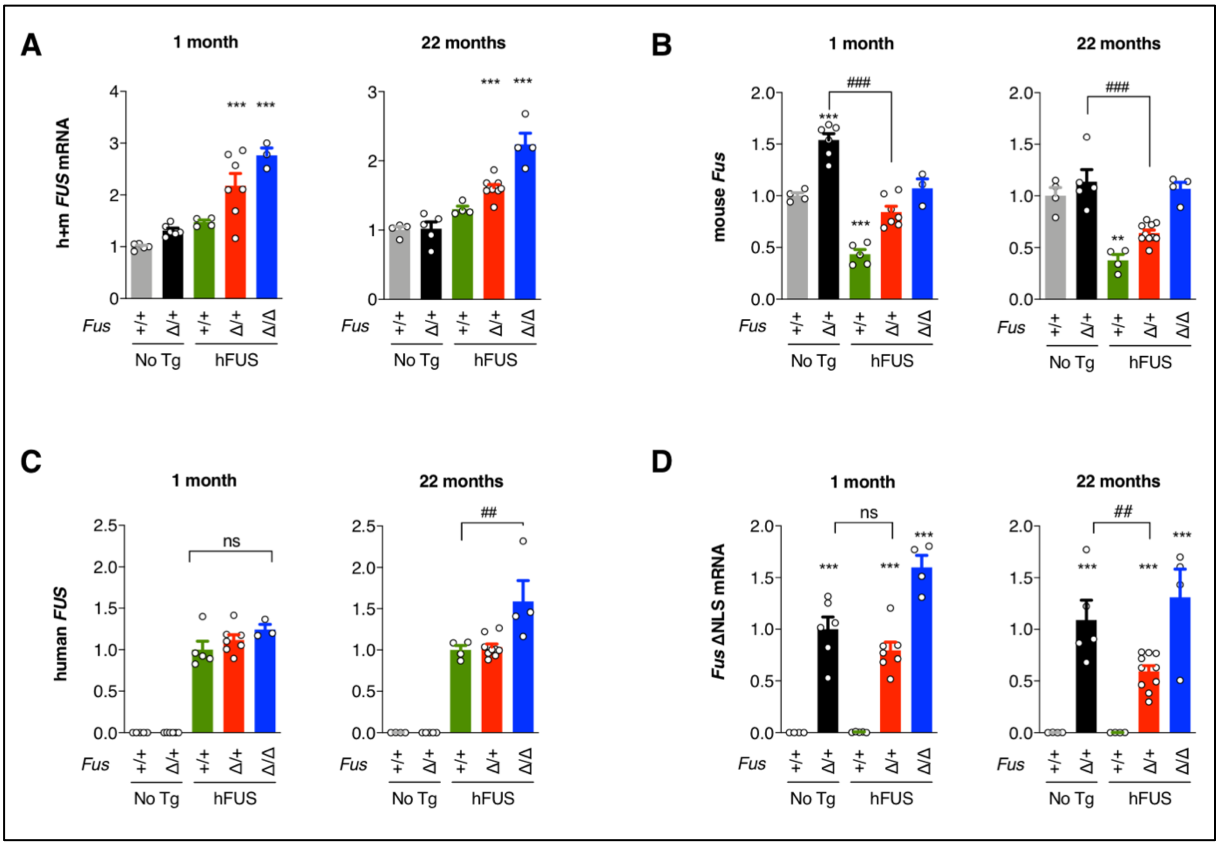

Supplementary Figure 2: hFUS transgene downregulates endogenous *Fus* mRNA levels in the frontal cortex

A-D: RT-qPCR results for total (human+ mouse mRNA encoding FUS (A), endogenous mouse *Fus* mRNA (B), human FUS transgene (C) and mutant *Fus* mRNA carrying the ΔNLS mutation in spinal cord at 1 month of age (left) or 22 months of age (right). Note that the hFUS transgene decreases expression of endogenous *Fus* gene and leads to decreased expression of mutant *Fus* mRNA at 1 and 22 months of age in the frontal cortex.

N = 4-8. ****p* < 0.001 vs $Fus^{+/+}$, ##, p<0.01, ###, p<0.001 vs indicated genotype by ANOVA followed by Tukey.

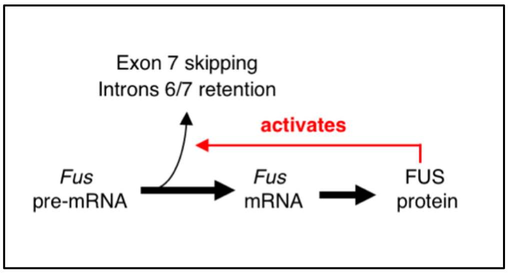

Supplementary Figure 3: Scheme depicting autoregulatory pathway of FUS expression

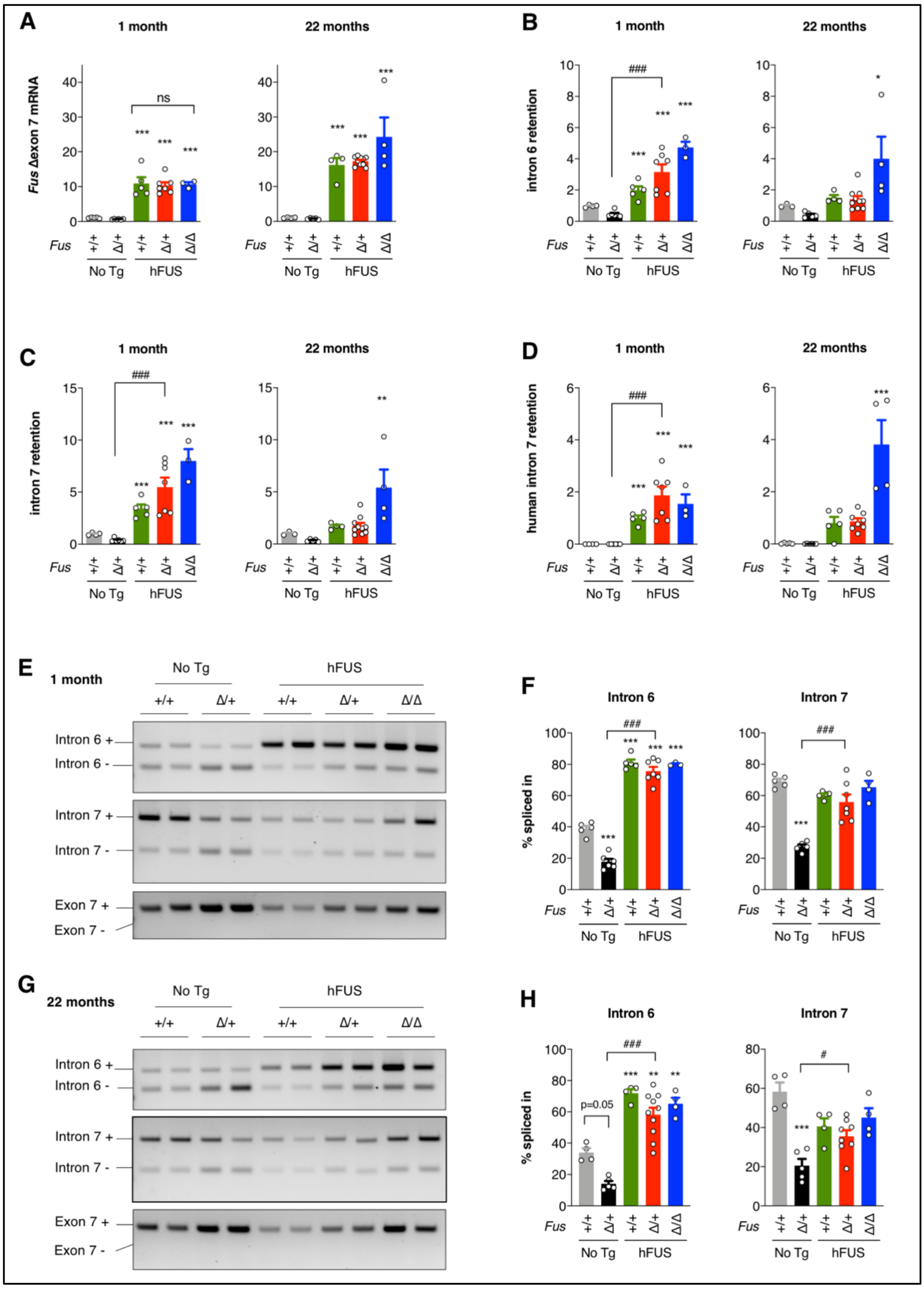

Supplementary Figure 4: hFUS transgene activates autoregulatory splicing in *Fus*$^{\Delta NLS/+}$ frontal cortex

A-D: RT-qPCR results for endogenous *Fus* mRNA deleted of exon 7 (A), endogenous *Fus* mRNA retaining intron 6 (B), endogenous *Fus* mRNA retaining intron 7 (C) and exogenous *FUS* mRNA retaining intron 7 (D) in frontal cortex at 1 month of age (left) or 22 months of age (right). Note that the hFUS transgene activates autoregulatory exon 7 skipping as well as retentions of introns 6 and 7 in endogenous mRNA and retention of intron 7 in exogenous mRNA at 1 and 22 months of age.

N = 4-8. **$p < 0.01$, ***$p < 0.001$ vs *Fus*$^{+/+}$, ###, p<0.001 vs indicated genotype by ANOVA followed by Tukey.

E-H: Representative gel electrophoresis of RT-PCR assays identifying RNA species with or without intron 6 retention (E, G, upper panel), with or without intron 7 retention (E, G, middle panel), or with or without exon 7 skipping (E, G, lower panel) in frontal cortex at 1(E) or 22 (G) months of age. We did not detect exon 7 skipped mRNA using these assays. Panels F and H show the percentage of intron 6 or 7 retention (intron + band intensity divided by the sum of intensities of intron + and intron – bands, multiplied by 100), for 1 month (F) or 22 months (H) of age.
N = 4-8. ** $p < 0.01$, *** $p < 0.001$ vs $Fus^{+/+}$, ##, p<0.01, ###, p<0.001 vs indicated genotype by ANOVA followed by Tukey

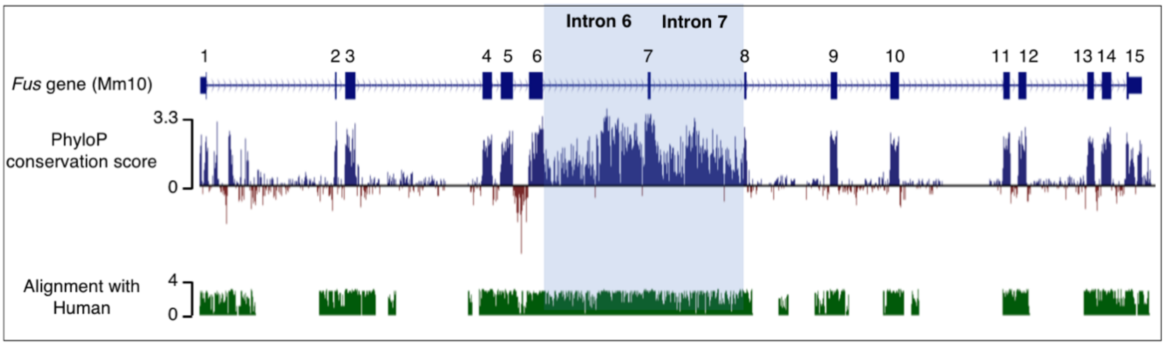

Supplementary Figure 5: conservation of introns 6 and 7 of the *Fus* gene
Genome browser snapshot of the mouse *Fus* gene, showing exon-intron structure (upper panel), PhyloP conservation score (middle panel) and mouse to human conservation (lower panel). Introns 6 and 7 are indicated by the shaded area. Note the extreme conservation of both introns, at levels similar to exon sequences.

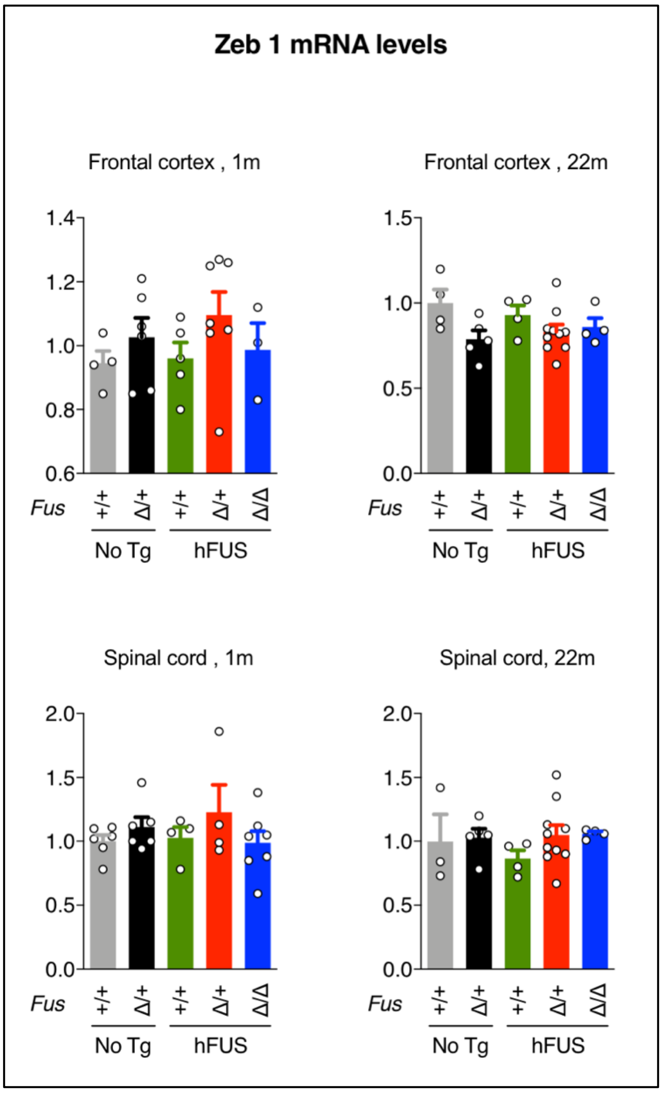

Supplementary Figure 6: *Zeb1* expression in Fus$^{\Delta NLS/+}$ spinal cord and frontal cortex
RT-qPCR results for *Zeb1* mRNA in frontal cortex (upper row) and spinal cord (lower row) at 1 month of age (left) or 22 months of age (right).
N = 4-8. No significant change in mRNA levels is observed. ANOVA followed by Tukey.
